# Nanoplastics Toxicity Specific to Liver in Inducing Metabolic Dysfunction—A Comprehensive Review

**DOI:** 10.3390/genes14030590

**Published:** 2023-02-26

**Authors:** Shoumi Haldar, Nounenuo Yhome, Yuvashree Muralidaran, Senthilkumar Rajagopal, Prabhakar Mishra

**Affiliations:** Department of Biotechnology, School of Applied Sciences, REVA University, Bengaluru 560064, Karnataka, India

**Keywords:** nanoplastics, hepatic glucose metabolism, lipid peroxidation, metabolic dysfunction, gut-liver axis

## Abstract

Plastic pollution in the world is widespread and growing. The environment is swamped with nanoplastics (<100 nm), and the health consequences of these less visible pollutants are unknown. Furthermore, there is evidence that microplastics can release nanoplastics by digestive disintegration, implying that macroplastic exposure can cause direct and indirect disease via nanoplastics. The existence and impact of nanoplastics in numerous tissues from invertebrates to larger vertebrates that consume significant amounts of plastics were investigated, and histopathological techniques were utilized to determine physiological reactions and inflammation from the plastics. Nanoplastics enters an organism through the respiratory and gastro-intestinal tract where they accumulate into the liver through blood circulation via absorption, or epidermal infiltration. It is stated that macroplastics can cause damage directly at the site of exposure, whereas nanoplastics can influence the liver, causing subsequent damage to other organs. Multi-organ dysfunction is brought on by liver changes, and nanoplastics can readily enter the gut-liver axis and disturb the gut microflora. By exploring the literature and summarizing the research that has been published to date, this review article reveals the deleterious effect and mechanisms of nanoplastics on the pathophysiological functions of the hepatic system.

## 1. Introduction

The global production of plastics has increased immensely over the past seven decades [1]. Globally, plastic pollution is a concern. Of the 9.2 billion tons, plastic manufactured between 1950 and 2017, almost 7 billion tons were wasted. About 3% of all plastic generated annually is thought to accumulated in the ocean. Up to 2015, 6.3 billion tons of the total was converted to waste. Only 9% of that used plastic is recycled, while the remaining 12% is burned. Since plastic does not dissolve, the remaining 79% are dumped in landfills or the environment, where they persist indefinitely in some shape or form [2]. Eighty-one out of 123 species of marine mammals are known to have eaten or become entangled in plastic. An estimate states that each year, 100,000 marine mammals die as a consequence of plastic contamination, and between 400,000 and one million people every year in developing nations pass away as a result of disorders and accidents related to improper waste management [3,4]. Due to the action of biodegradation, physical erosion, and oxidation, plastics present in the environment can be broken down into smaller particle sizes which can be macroplastics (>5 µm), microplastics (MPs) (1 µm–5 µm), and sizes between 1–100 nm are nanoplastics (NPs) [5]. Some of the common nanoplastics commonly used include polystyrene, polyethylene, polyvinyl chloride, polyamide, polyethylene, etc. [6]. Depending on the shape, size, chemical composition, surface chemistry, porosity, and concentration, nanoplastics pose several implications mostly for liver and kidneys inducing morphological as well as functional changes [7,8]. Studies have found that nanoplastics when entered inside the body gets accumulated in different organs like liver, lungs, guts, kidney, brain, thereby inducing several toxic damages [9,10].

The liver is one of the main metabolic organs that control the various metabolism pathways connecting to different organs and tissues and the major detoxifying organ in the human body [11,12]. The liver plays a major role in maintaining the body’s energy by controlling various pathways involves in glucose metabolism [13]. In liver, glucose is stored as glycogen and is the only source of blood glucose [14]. It also delivers glucose to different parts of the body and serves as the main site for gluconeogenesis [15]. The protein oxidation in the liver provides most of the energy required in the liver [16]. The protein metabolism involves the reaction of protein with water to form amino acids and dipeptides [17], where the amino acids are further broken into keto acids and ammonia [18]. In addition, urea production through the urea cycle takes place only in the liver in the human body [19]. The liver is also the main site for the metabolism of toxic chemicals [20,21]. 

Nanoplastics have been found to be associated with overproduction of reactive oxygen species in cells. NPs inside cells induced changes in different metabolic activities such as the TCA cycle, oxidative phosphorylation, degradation of fatty acid, metabolism related to amino acids and carbon [22,23]. Various metabolic pathways involving enzymes and proteins, biomarkers in the TCA cycle, amino acid, lipid, and nucleotide metabolism are either downregulated or upregulated depending on the concentrations and interaction with NPs [24]. Exposure to NPs also causes DNA damages [7,8]. Exposure to NPs leads to liver damage causing lipid peroxidation and oxidative stress [10]. NPs can enter cells, thereby causing mitochondrial damage in the liver cells leading to improper functioning of cells and tissues [25]. Studies had shown an increased production of mROS due to inhibition of electron transport chain (ETC) when exposed to NPs at high concentration [9,26].

Experimental studies show decreased activities of several enzymes, oxidative stress in fish, mice, and liver cell lines [27]. Alteration in lipid, amino acid, and energy metabolism, inflammation, low liver weight, and lipid accumulation were also observed in a mouse model [28]. A study in fish and mouse indicates that with the toxicity the of gut due to microplastics and nanoplastics, they enter the liver, causing liver tissue damage and chronic inflammation [29,30]. Current studies on the effect NPs on human cells and tissues need more experimental and research work, and we are still at an initial phase in understanding the toxicity related to nanoplastics in humans.

## 2. Pathways Involved in Liver Metabolism

NPs of diameter <100 nm have had particular attention in the science field and are commonly used in studies, because they are internalized by cells more than larger particles [31]. Evidence has indicated that the liver and kidneys are major accumulation sites, as well as vital organs for the metabolism and clearance of nanomaterials [32]. The accumulation of polystyrene nanoplastics (PS NPs) in the liver and kidneys of mice has been documented, and exposure to NPs induced evident morphological and functional changes in these two organs, suggesting the importance of investigating the impact of NPs on liver and renal cells [33,34,35].

**Figure 1:**
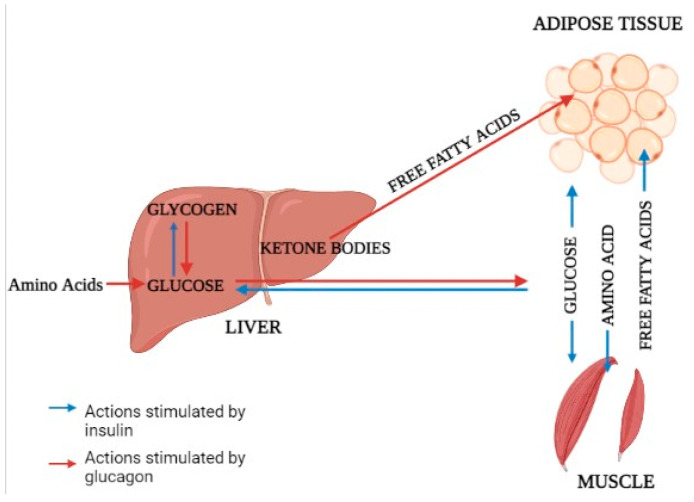
Interaction between liver and muscle tissues.

The majority of total glucose disposal happens in insulin-independent tissues, with roughly 50% occurring in the brain and 25% occurring in the splanchnic region (liver and the gastrointestinal tissues) [36]. The release of glucose from the liver closely matches glucose use, which averages around 2.0 mg/kg/min [37]. The majority of glucose elimination in the body happens in muscle tissue after consuming a glucose-containing meal [38]. Glucose homeostasis is dependent on three interconnected processes: pancreatic insulin secretion, stimulation of glucose uptake by splanchnic (liver) and peripheral tissues, and inhibition of hepatic glucose output [39]. Adipose tissue accounts for only 4%–5% of total body glucose elimination, which plays a critical role in maintaining whole body glucose homeostasis [40]. Pathways involved in liver metabolism related to muscles and adipose tissue are also mentioned (Figure 1).

### 2.1. Glucose Metabolism

In humans, muscle is the principal location of glucose excretion. Once in the cell, glucose can be converted to glycogen or enter the glycolytic pathway [41]. Because obesity and diabetes are the two most prominent risk factors for the development of liver disorder, the presence of one or both of them can explain some of the peripheral insulin resistance IR [42,43]. Glucose disposal is reduced by about 50% in nondiabetic patients compared to normal people, a level comparable to type-2 diabetes mellitus (T2DM) [44]. The fact that there is no difference in glucose disposal between normal and overweight patients shows that the problem is not solely connected with improper glucose regulation and/or excess fat accumulation [45]. From fatty liver to nonalcoholic steatohepatitis (NASH), glucose utilization in muscle deteriorates progressively, and hyperinsulinemia are not secondary to a decrease in hepatic insulin extraction [46,47,48].

When paired with the clamp, indirect calorimetry provides the rate of non-oxidative glucose disposal (i.e., glycogen synthesis) as the difference between whole-body glucose absorption and glucose oxidation [49]. Insulin-stimulated glycogen synthesis is reduced in insulin-resistant states such as obesity, diabetes, and the combined obesity–diabetes syndrome [50]. 

### 2.2. Glucose Output

After an overnight fast, a healthy person’s liver produces glucose at a rate sufficient to meet the needs of the brain [51]. The variability of basal human glucose output (HGO) in nondiabetic persons is mostly determined by the amount of lean body mass and the degree of peripheral insulin use [52,53,54]. HGO is suppressed by insulin released into the portal vein following glucose or meal consumption. Hepatic IR occurs when the liver fails to detect this signal [54]. The glucose produced by the liver can come from glycogenolysis or gluconeogenesis [55]. Insulin suppresses HGO either directly through the hepatic insulin receptor or indirectly by reduced production of gluconeogenic substrates (e.g., alanine, lactate, glycerol, and free fatty acids) in both muscle and liver [56,57].

### 2.3. Lipolysis and Lipid Oxidation

Aside from muscle and liver, adipose tissue is the third metabolically significant site of insulin action. Whereas insulin-stimulated glucose disposal in fat tissue is minor in comparison to that in muscle, regulation of lipolysis with subsequent release of glycerol and free fatty acids (FFAs) into the bloodstream has significant implications for glucose homeostasis [40,58]. Increased availability and utilization of FFAs aid in the development of skeletal muscle IR by inhibiting substrate oxidation competitively [59]. According to recent 1H nuclear magnetic resonance studies, an increase in intracellular fatty acid metabolites impairs IRS-1 tyrosine phosphorylation, resulting in decreased PI3-kinase activity and glucose transport [60]. Free fatty acids (FFAs) stimulate key enzymes and provide energy for gluconeogenesis, while glycerol released during triglyceride hydrolysis acts as a gluconeogenic substrate. 

When insulin levels are high, hepatic FFA esterification takes precedence over oxidation until the intracellular long chain acyl coenzyme is depleted. The concentration raises high enough to overcome the inhibitory effect of malonyl-coenzyme-A on carnitine palmatoyl transferase. Both fatty acid esterification and oxidation will be improved as a result. Normal subjects with as little as 10% liver fat have a reduced ability of insulin to suppress serum FFAs [61]. Similarly, higher values of plasma FFAs during the clamp characterize the greater IR in T2DM with fatty liver and hepatic steatosis correlated with basal and insulin-stimulated plasma FFAs. Fasting plasma FFA levels are also linked to muscle CT attenuation indices, which are a measure of muscle fat content [62,63].

### 2.4. Fatty Acid Metabolism and Energy Supply

The Krebs cycle oxidizes acetyl-coenzyme A (CoA), resulting in reduced forms of nicotinamide adenine dinucleotide (NADH) and reduced flavine-adenine dinucleotide, which transport electrons to the MRC [64]. AMPK promotes glucose and fatty acid oxidation while also activating PGC-1a [64,65]. PGC-1a interacts with peroxisome proliferator-activated receptor a (PPARa) to promote the production of numerous fatty acid-metabolizing enzymes, including carnitine palmitoyltransferase 1 (CPT1) and acyl-CoA dehydrogenases, hence enhancing fatty acid b-oxidation in mitochondria [66,67,68,69]. PGC-1a also increases the expression of TFAM by inducing its expression and binding to NRF1 [70,71,72]. NRF1 and TFAM regulate mtDNA transcription and replication, respectively, while NRF1 controls the expression of nuclear DNA-encoded MRC proteins. PGC-1a increases mitochondrial mass as well as oxidative phosphorylation capacity in the mitochondria [73,74].

### 2.5. Mitochondrial Damage

The TCA cycle is essential for cellular energy metabolism because it provides energy for cellular respiration [75]. In one study, NP-treated L02 cells had higher levels of some endogenous indicators of the TCA cycle, such as malate, and lower levels of others, such as fumarate. The effects of 80 nm NPs on mitochondrial activities and metabolic pathways in normal human hepatic (L02) cells were studied. NP did not cause widespread cell death. However, transmission electron microscopy analysis revealed that the NPs could enter the cells and cause mitochondrial damage, as evidenced by increased production of reactive oxygen species in the mitochondria, changes in the mitochondrial membrane potential, and suppression of mitochondrial respiration. In NP concentrations as low as 0.0125 mg/mL, these changes were observed. Overproduction of mROS has been identified as one of the primary causes of mitochondrial damage [76,77]. In L02 cells, NP administration boosted mROS generation in a dose-dependent manner, with effects detectable even after low NP concentrations [78].

Purine metabolism is one of the primary metabolic processes involved in cellular ATP synthesis, and changes in ATP can result in changes in metabolic phenotype [79]. It was shown that ATP concentrations in L02 cells were dramatically reduced in a dose-dependent manner after NP administration, which is consistent with the reported changes in ATP synthesis by the mitochondrial ETC. The contents of most downstream endogenous indicators of the purine metabolism pathway decreased. These findings indicate that this NP therapy drastically reduced the purine pathway. The considerable elevation of GSH in L02 cells caused by NP administration, which occurred in a dose-dependent manner, can be regarded as an adaptive response because it would have strengthened the antioxidant defense mechanism [80,81].

### 2.6. Protein and Urea Metabolism

Among the three key dietary categories of protein, lipids, and carbohydrates, protein metabolism is crucial for the production of albumin and prothrombin as well as for the detoxification of ammonia. When these mechanisms are addressed in nutrition treatment for liver cirrhosis, they affect the development of hepatic encephalopathy and the hepatic functional reserve, leading to an imbalance in branched-chain amino acid (BCAA) insufficiency, reduced albumin synthesis, and elevated blood ammonia concentrations [82]. The primary reason of BCAA shortage in liver cirrhosis patients is an increase in ammonia metabolism in the skeletal muscles [83]. About half of the ammonia in healthy individuals is digested in the liver’s urea cycle, while the other half is processed in the skeletal muscles’ glutamine producing system. The liver produces glutamine and uses it to detoxify ammonia, with the urea cycle serving as the primary mechanism. In liver cirrhosis, zinc deficiency results in decreased urea cycle activity. When there is liver cirrhosis, the urea cycle is less functional due to zinc shortage, which reduces the ability to detoxify ammonia [82].

### 2.7. Ethanol Metabolism 

There are at least three different categories of ethanol’s metabolic effects: those brought on by changes in metabolite pools and cofactors brought on by the ethanol’s metabolism, those brought on by neuroendocrine issues brought on by intoxication, and those brought on directly by the pharmacological effects of ethanol on particular cells and processes. These numerous sorts of effects have varying degrees of contribution in practically every significant domain of metabolism. The apparent discrepancies about the metabolic effects of ethanol frequently result from variations in experimental setups that affect how much each of these components contributes [83,84].

The first category of effects, those brought on by ethanol metabolism, have received the greatest attention. The primary result is a rise in the NADH: NAD+ ratio in the mitochondria and cytoplasm of the liver cell. This has an impact on the availability of pyruvate and oxaloacetate, affecting the oxidation of fatty acids and other substrates, gluconeogenesis, and carbohydrate consumption in the mitochondria. Additionally, numerous additional NAD+-dependent processes involved in the metabolism of amino acids, biogenic amines, glycerol, carbohydrates, porphyrins, and molecules of other types are directly impacted by the change in nucleotide ratio. Finally, the liver’s high output of acetate, lactate, and lipids, together with the lower levels of acetaldehyde, have indirect impacts on the metabolism of other organs [85,86,87].

Less research has been done on the second category of effects, those that are determined by the level of intoxication. Hepatic glycogenolysis caused by high doses of ethanol is mediated by sympathetic and adrenomedullary responses. Additionally, it is likely that mobilization of fatty acids from peripheral adipose tissue contributes to the development of hepatic steatosis following a single high dosage of ethanol. Much of the variability in ethanol metabolism and its impact on other drug metabolism may be attributed to hypoxia and disturbances in blood flow through various organs [88].

The third class of effects, those brought on by ethanol’s direct pharmacological action, have received the least attention despite the possibility that they might have a significant impact. Alcohol may inhibit the active transport of amino acids in the liver, gastrointestinal tract, and elsewhere, according to very limited data. Additionally, it could directly affect renal tubular transport mechanisms, the permeability of mitochondrial and cell membranes, and the processes involved in the production and exocytosis of lipoproteins in the liver. All of them would have significant metabolic repercussions if they were shown to be true [89,90].

The effects of the first type prevail at low concentrations of ethanol in bodily fluids, whereas those of the second and third kinds become increasingly significant at greater concentrations due to the unusual kinetics of ethanol oxidation in vivo. The metabolic effects of ethanol must be explained or predicted in light of this diversity as well as the aggravating aspects of nutritional imbalance and hepatic disease that may develop after prolonged ethanol use [90].

## 3. Impact of Nanoplastics on Liver Metabolism Causing Multi-Organ Dysfunction

**Figure 2:**
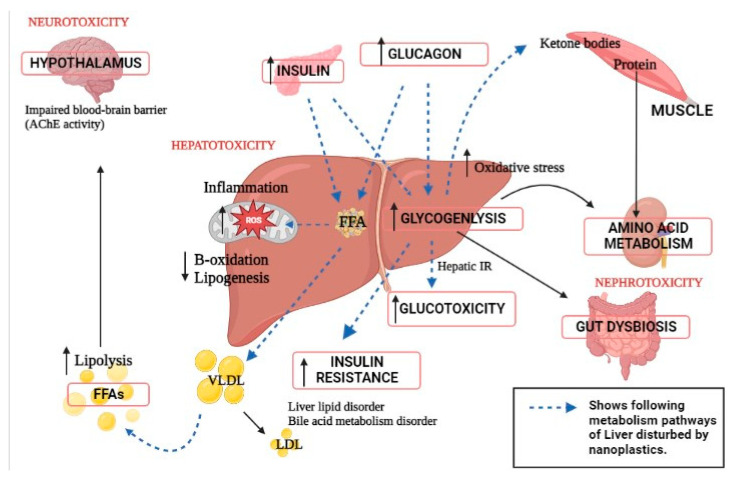
Disturbed metabolism pathways of the liver and neighboring organ dysfunction due to nanoplastics exposure.

The liver, a central metabolic organ, alters lipid metabolism in response to harsh environmental conditions [91]. Pathways involved in liver metabolism and neighboring organ dysfunction due to nanoplastics toxicity are presented (Figure 2). After being exposed to 100 ppm of PS-NPs, Lu et al. (2016) discovered that huge volumes of lipid droplets developed in the liver of the zebrafish *Danio rerio* [92]. As reported, it showed greater hepatic inflammation. According to the findings, dietary exposure to PS-NPs increased oxidative stress and interfered with lipid metabolism. Furthermore, after NP exposure, the crude lipid content of turbot *Scophthalmus maximus* and black rockfish *Sebastes schlegelii* livers increases dramatically [93,94]. An in vitro experiment revealed that NPs bind to apolipoprotein A-1 in fish serum, limiting lipid utilization [95,96]. According to a study, after PS NPs exposure, the liver TG and lipid content were considerably greater than in the control group. In fed fish, mRNA expression of the lipid synthesis-related gene *fas* and lipid transport-related genes such as *cd36* and *fatp1* increased considerably [97]. The mRNA expression of lipid catabolism-related genes such as *ppar* and *aco* increased initially and then declined as PS NP concentrations increased. PS NPs were found to boost the expression of genes involved in lipid production and catabolism in hepatocytes in the short term [98]. The trans-generational effect showed fabp10a expression in larval livers in a dose- and size-dependent manner. The 50 nm PS-NPs of 0.1ppm concentration raised fabp10a expression in the larval liver by 21.90%. These impacts’ potential mechanisms are dependent on their distribution and the formation of reactive oxygen species in the larvae. NPs also stimulate steroid hormone biosynthesis in zebrafish larvae, which may result in immune-related disorders [99,100].

Brandts et al. (2018) demonstrated that transcriptional machinery is activated by NPs in the liver of *D. labrax*, resulting in enhanced expression of lipid-related genes [101,102]. *D. Labrax* were treated with ~45nm NPs at various concentrations (0.02 mg/L, 0.2 mg/L, 2 mg/L, and 20 mg/L) for 96 h. It was shown to have inflammation and hepatocyte proliferation and also to have reduced the glucose metabolism. Lipids’ main function in fish is to store and provide energy in the form of adenosine triphosphate (ATP) via oxidation of fatty acids, which is a key source of energy for many fish species [103,104]. This oxidation occurs in cellular organelles such as mitochondria and peroxisomes and is catalyzed by a variety of enzymes [105]. As essential regulators of lipid metabolism, peroxisome proliferator-activated receptors (PPARs) respond to fatty acid signals [106,107]. The expression of genes involved in innate immune function in *D. labrax* was also assessed. After 96 h of exposure, the levels of *ppara*, *pparγ*, and *nd5* were affected but *IL1*, *IL6*, *tnf*, and *IL10* were unaffected, indicating that NPs did not directly affect *D. labrax*’s immune system on a molecular level, indicating no cellular stress was significant [108].

An experiment was conducted to test trans-generational toxicity. Mice were treated to PS-NPs of size 100 nm at varied concentrations (0.1, 1, and 10 mg/L) during gestation and lactation. Results showed that PS-NPs exposure during pregnancy and breastfeeding decreased birth and postnatal body weight in offspring mice. Furthermore, high-dose PS-NPs lowered liver weight, produced oxidative stress, infiltrated inflammatory cells, increased pro-inflammatory cytokine production, and disrupted glyco-metabolism in male offspring mice liver [109]. Another study demonstrated that PS-NPs may worsen chronic hepatitis in mice by interfering with hepatic lipid metabolism. Furthermore, hepatic tissue from PS-NPs-treated HFD animals had significantly decreased superoxide dismutase (SOD) activity, confirming the oxidative stress caused by PS-NPs. PS-NPs exposure significantly increased the inflammatory response in the liver, as demonstrated by increased infiltration of Kupffer cells (KCs) and increased expression of pro-inflammatory associated markers. Results showed that offspring’s birth and postnatal body weight decreased after the mother was exposed to PS-NPs during pregnancy and lactation. Furthermore, in the liver of male progeny mice, high-dosage of PS-NPs decreased liver weight, induced oxidative stress, induced inflammatory cell infiltration, increased pro-inflammatory cytokine production, and disrupted glyco-metabolism [109].

Another study was conducted where zebrafish were fed with 42 nm PS-NPS (i.e., dietary exposure) at various concentration (90, 45, and 120 mg/mL). Nanoplastics have been demonstrated to cause oxidative stress, alterations in locomotor activity, and developmental problems in zebrafish [110]. Higher levels of reactive oxygen species ROS (i.e., hydrogen peroxide and organic peroxides) can be caused by an imbalance between the synthesis and detoxification of ROS, which causing oxidative stress, cellular damage, and apoptosis. In F0 adults, maternally or co-parentally exposed larvae showed lower GR activity, thiol levels, and glutathione metabolism at 96 hpf (hours post fertilization). Similar alterations were also visible in F1 [111].

In a similar investigation, zebrafish were treated with 80 nm of PS-NPs at different concentrations of 0.01, 0.1, 1, 5, and 10 mg/L for 24 h. Furthermore, co-exposure increased mortality, accelerated voluntary movements, increased hatching rate, and lowered heart rate considerably. Hepatotoxicity tests found that zebrafish larvae exposed to the mixture had a darker/browner liver color, atrophied liver, and increased hepatotoxicity. In addition to increased ROS generation, co-treatment resulted in decreased expression of the antioxidant gpx1a gene and increased expression of cyp1. Additionally, the genes AChE and chrn7α, are linked to neuro-central development and were significantly downregulated. When compared to the PS-NPs single exposure, the results demonstrated a change in yolk membrane structure, as well as particle bio-accumulation in the intestine of zebrafish larvae [112]. A summary of the effects of accumulation of NPs in the liver of various model organisms is shown in Table 1.

In another investigation, the embryo was filled with 3 nL of NPs (20 nm) for 4 hpf. The results revealed a significant rise in total cellular death. In the brains of 33% of the zebrafish larvae that were investigated, cumulative bioaccumulation of NPs was found. As shown, NPs that are injected into the yolk sac can go to the brain, where they can bioaccumulate and lead to physical abnormalities. In the areas of the zebrafish larvae’s brain where NPs concentrate, oxidative DNA damage has also been caused. According to the findings, injecting NP caused a 27% increase in mortality and a little delay in zebrafish embryo hatching [121].

Another study reveals that from their embryonic to larval phases for 4 h after fertilization, 50 nm and 100 nm NPs were employed to track their accumulation processes. The plasma membrane’s structural integrity is harmed by lipid peroxidation, which is caused by elevated ROS levels in cell membranes [122]. The expression of the liver-specific fatty acid binding protein fabp10 increased in response to NP exposure, increasing the risk of hepatic inflammation [123].

In another experiment, human liver cell lines (Lo2) were exposed to 80 nm nanoparticles at concentrations of 0.125 and 0.25 mg/mL. The results showed that high NP concentrations caused a reduction in cell viability. A dose-dependent increase in mROS generation was seen after NP administration. The outcomes of metabolic pathways demonstrated that NP exposure altered the metabolism of nicotinate and nicotinamide in L02 cells. The tricarboxylic acid (TCA) cycle, glutathione (GSH) metabolism, and purine metabolism were all negatively influenced by the NPs exposure, as well as the urea cycle and electron transport chain (ETC) in the mitochondria [8].

Medaka was treated with 100 nm of PSNPs at a concentration of 10 mg/cm^3^ which resulted in the alteration in the enzyme activities as well as antioxidant activity such as SOD and CAT activity. Exposure to a high concentration of NPs inhibited antioxidant enzyme activity and produces toxic effects due to increased ROS levels [124].

## 4. Alteration in Gut–Liver Axis Due to Nanoplastic Toxicity 

The portal vein, which carries blood from the human digestive system to the liver and establishes the foundation for a strong bidirectional interaction between these two critical organs, connects the human liver and stomach. The liver participates via bile acid production and the facilitation of some parts of the human immune response via the portal vein, which transports different metabolites from the digestive tract to the liver [125].

There is evidence that gut microbiota and liver are related as a result of environmental pollutants. Mice were exposed to 1 mm polystyrene microplastics at a concentration of 10,000 g/L in one experiment. The main metabolic alterations in the liver after one week of microplastic exposure may be divided into two categories. The mice provided protection against the oxidative damage in the first section. In the second section, the mice’s gut–liver axis was disturbed, which increased in developing insulin resistance. After one week of exposure to microplastics, metabolites were altering, which raises the chance of developing diabetes. The regulation of these metabolites has significant effects on insulin resistance and the gut–liver axis metabolism. The gut–liver axis was linked to two elevated metabolites, 4-guanidinobutyric acid and CDP-choline. Mice showed significance for experiencing oxidative stress and building up resistance to it after one week of exposure to microplastics. The exposure to microplastics in mice disrupted the gut–liver axis based on information on intestinal microbiota and differently expressed metabolites [126]. In another investigation, a dose of 1 mg/L of 5 m PS-MPs was exposed to chickens. The findings showed that liver injury was caused by disruption of the intestinal flora through the intestinal liver axis, and that pathogenic bacteria and their byproducts were implicated in liver damage through translocation of the gut–liver axis. According to metabolomics and transcriptome data, apoptosis and abnormal lipid metabolism were the primary contributors to the liver damage produced by PS-MPs [126].

In another study, male marine medaka were given treatments with 2 m and 200 m PS-MPs at concentrations of 0.3 g/mg and 3 g/mg, and the findings demonstrated growth suppression and inflammation, and caused oxidative stress, which resulted in a noticeably higher level of SOD (Superoxide dismutase) activity, MDA (Malondialdehyde), and CAT (Catalase) activity. The intestinal bacteria of *Enchytraeus crypticus* were altered by MP exposure, with an increase in *Amoebophilaceae* and a decrease in *Isosphaeraceae*, *Rhizobiaceae*, and *Xanthobacteraceae*. The presence of many bacteria, which eventually affects the gut–liver axis, is also substantially associated with the metabolic pathways related to glycerolipid metabolism, energy/carbohydrate metabolism, amino acid metabolism, cholesterol, and stress. Fish liver injury can also result through intestinal–liver axis disorders rather than microplastics’ direct effects. The host metabolic changes that modify the structure or functional capacity of the gut microbiome and liver were shown to be related to the changes in gut microbial composition in this study [127].

In a continuation of the previous section, it is seen that nanoplastics show toxicity to other organs directly or indirectly. Based on our findings, we can conclude that the micro-plastics exposure of organisms shows disturbance in the gut–liver axis. As nanoplastics are smaller in size than microplastics, they have a high surface volume and are more susceptible to cause toxicity. Hence, it is hypothesized that nanoplastics can easily invade the intestinal barrier and affect the gut–liver axis to the metabolism pathways. Therefore, there is a scope for future studies and analysis on the gut–liver axis towards nanoplastic toxicity. 

## 5. Conclusions and Future Perspective

With the increased usage of plastic products and human exposure, people have progressively begun to pay attention to the negative consequences of plastic products. The liver is the body’s largest organ, responsible for cleansing and metabolism, and it performs numerous critical functions. Researchers are paying increasing attention to the harmful effects of NPs on the liver. According to the findings, many studies focus on the toxic effects of NPs on the liver, and NPs have unique characteristics that have stronger effects in inducing the production of ROS (Reactive Oxygen Species) in the liver and the development of oxidative stress and inflammation, and more research on the effects of NPs should be conducted. In contrast to the well-studied toxicity of nanoplastics, which has been extensively reviewed, the toxicity caused by the variety of NPs has yet to be properly examined.

Further research should also take into account how stable plastic particles react when they are combined with other environmental pollutants because this might change how organisms interact with them. Investigating whether or if the pollutants that have been adsorbed onto NPs are desorbed inside the organism, and whether this results in increased or decreased egestion, would also be essential. It is crucial to take into account how changing the species of pollutants or the shape of plastic particles might affect the mixture’s overall toxicity.

## Figures and Tables

**Table 1:** The effects of accumulation of NPs in the liver of various model organisms.

Model Organism	Particle Size and Concentration	Mode of Exposure	Exposure Time	Consequences	References
Female mice (*Mus musculus*)	42 nm 10, 50 μg/mL	Tail vein injection	15 days	Increased hepatocyte bi-nucleation Fatty acid degeneration Severe perilobular steatosis	[113]
Fish (*Vertebrata*)	60 nm 5 mg/L	Water	7 days	Aggregated and condensed nuclei.	[114]
Zebrafish (*Danio rerio*)	70 nm 20, 200, 2000 μg/L	Water	3 weeks	Necrosis Infiltration Fat droplets observed in hepatocytes.	[115]
Male C57 mice (*Mus musculus*)	100 nm 0.1, 1 mg/L	Water	60 days	Hepatocellular edema and vacuolar degeneration, enlarged nucleus, cell dikaryon, inflammation of portal areas	[116]
Juvenile groupers	100.86 ± 7.15 nm 300, 3000 μg/L	Water	14 days	Hepatocyte vacuolization	[117]
Little yellow croaker (*Larimichthys polyactis*)	190 nm 1 mg/L	Feeding	8 days	Necrosis Decrease in tissue density	[118]
Goldfish (*Carassius auratus*)	250 nm 0, 0.05, 0.5, 5 mg/L	Water	28 days	Necrosis, Cellular swelling Hemorrhage	[119]
Wistar male rats (*Rattus norvegicus*)	25, 50 nm 1, 3, 6, 10 mg/kg bw/day	Oral gavage	5 weeks	NPs accumulated in whole body	[120]

## Data Availability

Not applicable.
